# Inherited Retinal Diseases

**DOI:** 10.3390/ijms232113467

**Published:** 2022-11-03

**Authors:** Tamar Ben-Yosef

**Affiliations:** Ruth and Bruce Rappaport Faculty of Medicine, Technion-Israel Institute of Technology, Haifa 3109601, Israel; benyosef@technion.ac.il ; Tel.: +972-4-829-5228

Inherited retinal diseases (IRDs) are a clinically and genetically heterogeneous group of diseases that cause vision loss due to abnormal development or due to the dysfunction or degeneration of the photoreceptors or the retinal pigment epithelium. They have a prevalence of approximately 1:1380 individuals, with 5.5 million people expected to be affected worldwide. The most common form of IRD is Retinitis Pigmentosa. Other forms include cone/cone–rod degeneration, Leber congenital amaurosis, inherited macular dystrophies, among others [1]. While in most IRD cases, the disease only involves ophthalmic manifestations (nonsyndromic), over 70 forms of syndromic IRDs have been described [2]. The most common form of syndromic IRD is Usher syndrome (see review by Fuster-Garcia et al., in this Special Issue) [3]). The aim of this Special Issue was to focus on the major challenges in the IRD field, including phenotypic characterization, molecular diagnosis, functional studies, and therapy.

IRD is the most genetically heterogeneous group of disorders in humans. It can be inherited as autosomal recessive, autosomal dominant, or X-linked. Mitochondrial and digenic patterns of inheritance have also been described. Since the identification of the first two IRD-causative genes back in 1990, *RHO* and *CHM* [4,5], an average of nine newly identified causative genes have been identified every year between the years 1990 and 2021, and to date, at least 280 genes have been implicated in IRD (RetNet at https://sph.uth.edu/Retnet/, accessed on 30 October 2022). However, this discovery phase has now reached a plateau (Figure 1), indicating that while some rare novel IRD genes may exist, the vast majority of IRD-causative genes have already been discovered. Nevertheless, the contribution of each of these genes to the overall prevalence of the disease is relatively small, and 65% of identified pathogenic variants are unique to a single individual [1]. Moreover, despite the wide application of Next-Generation Sequencing (NGS) on large IRD cohorts, reported mutation identification rates worldwide range between 50 and 76%. This is true not only for the cohorts analyzed by whole exome sequencing or targeted NGS (i.e., gene panels), but also for cohorts analyzed by whole genome sequencing [6,7,8]. Taken together, the conclusion is that most of the missing mutations are located in known IRD genes and are not yet identified, either due to technical limitations or to misinterpretation. The significant proportion of cases that remain unsolved (up to 30%) poses a challenge for both clinical and research aspects. Accurate, sensitive, and cost-effective genetic analysis of IRD patients therefore requires the combined development of efficient sequencing and bioinformatics tools as well as thoughtful analysis workflows. Moreover, functional evaluation is required to prove the pathogenicity of some of the identified variants. Excellent examples for such developments are provided and reviewed in this Special Issue [9,10,11,12,13,14].

In recent years, the IRD field has undergone dramatic changes, mainly due to the development of novel therapeutic strategies that are either non-gene-based (stem cell therapy and retinal implants), gene-based (gene therapy and targeted pharmacological agents), or mutation-based (translational read-through inducing drugs, antisense oligonucleotides; The latter is used in the study by Tomkiewicz et al. [15]). A large number of clinical trials targeting various IRD-related genes and mutations are currently ongoing. Comprehensive studies on the clinical spectrum and natural history associated with various IRD-causative genes and mutations are greatly important not only to define prognosis, but also to identify clinical trial endpoints [14,16,17,18,19,20,21,22,23].

Finally, the biological function and disease etiology of many of the genes associated with IRD are not fully understood. Studies addressing these questions while using various experimental systems (such as the ones by Aísa-Marín et al., Koch et al., and Remez et al. [24,25,26]) provide significant insights into mechanisms of normal retinal function and the types of cellular defects that lead to retinal degeneration. These findings will help in the prevention and development of new therapeutic strategies for retinal degeneration.

## Figures and Tables

**Figure 1:**
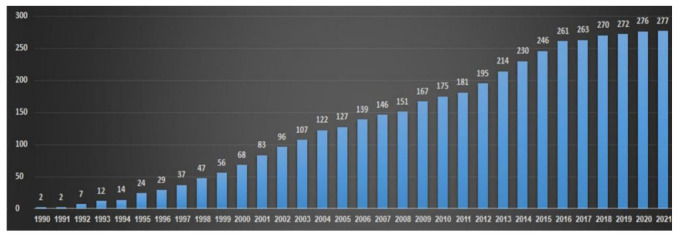
IRD-causative gene identification over the years. The chart shows the total number of known IRD-causative genes each year between 1990 and 2021.

## Abbreviations

IRD	Inherited Retinal Diseases
NGS	Next-Generation Sequencing
